# Targeted demethylation of the SLC5A7 promotor inhibits colorectal cancer progression

**DOI:** 10.1186/s13148-022-01308-5

**Published:** 2022-07-20

**Authors:** Yang Li, Baike Liu, Xiaonan Yin, Zhiyuan Jiang, Chao Fang, Na Chen, Bo Zhang, Lei Dai, Yuan Yin

**Affiliations:** 1 Department of Gastrointestinal Surgery, Guang’an People’s Hospital, Guang’an, 638500 Sichuan People’s Republic of China; 2 Department of Gastrointestinal Surgery, West China Hospital and State Key Laboratory of Biotherapy, Sichuan University, Chengdu, 610041 Sichuan People’s Republic of China; 3 State Key Laboratory of Biotherapy and Cancer Center, West China Hospital, Sichuan University and Collaborative Innovation Center for Biotherapy, Chengdu, 610041 Sichuan People’s Republic of China; 4 School of Pharmacy, Chengdu Medical College, Chengdu, 610500 People’s Republic of China

**Keywords:** Colorectal cancer, SLC5A7, Targeted demethylation, p53, TET1CD

## Abstract

**Background:**

SLC5A7 (solute carrier family 5 member 7), also known as choline transporter 1 (CHT1), is downregulated in colorectal cancer (CRC) and functions as a tumor suppressor. However, the mechanisms underlying the inactivation of SLC5A7 in CRC remain to be elucidated.

**Results:**

In the present study, two broad-spectrum demethylation agents (azacitidine and decitabine) employed to treat CRC cells significantly upregulated SLC5A7 expression. Further results based on the CRC cohort and TCGA database indicated that SLC5A7 promoter methylation inversely correlated with SLC5A7 expression, and the status of SLC5A7 promotor methylation showed a promising prognostic value for patients with CRC. Next, the dCas9-multiGCN4/scFv-TET1CD-based precision demethylation system was constructed, which could significantly and specifically promote SLC5A7 expression in CRC cells through sgRNA targeting the SLC5A7 promoter. Both in vitro and in vivo experiments demonstrated that targeted demethylation of SLC5A7 by dCas9-multiGCN4/scFv-TET1CD-sgSLC5A7 inhibited tumor growth by stabilizing p53 and regulating downstream targets.

**Conclusions:**

Collectively, DNA promoter methylation caused inactivation of SLC5A7 in CRC, and targeted demethylation of SLC5A7 might be a therapeutic target for CRC and other cancers.

**Supplementary Information:**

The online version contains supplementary material available at 10.1186/s13148-022-01308-5.

## Introduction

DNA methylation plays a pivotal role in gene regulation at the transcriptional level, and alterations in methylation regulate diverse pathological processes, including cancer initiation and progression [1–3]. Hence, targeting the modulation of DNA methylation could be a prospective approach in malignancy management [4]. Azacitidine (5-azacytidine, AZA) and decitabine (5-aza-2′-deoxycytidine, DAC) are two broad-spectrum DNA methyltransferase inhibitors (DNMTis) approved by the Food and Drug Administration (FDA) as DNA demethylation drugs for the treatment of myelodysplastic syndrome (MDS), leukemia, and other malignancies [5–7]. However, these broad-spectrum demethylation agents have some undesirable side effects, and the lack of specificity limits the utilization of AZA or DAC in clinical practice [8]. To achieve a more precise DNA demethylation, several systems such as zinc fingers (ZFs) [9], transcription activator-like effector (TALE) [10], and recently the CRISPR–Cas9 [11–13] have been applied for precise epigenome modification through linkage to epigenome-regulating reagents. A CRISPR-Cas9 system is mentioned consisting of dead(d)Cas9 fused to the catalytic domain of ten eleven translocation 1 (TET1CD), which is a member of the TET protein family [14]. The system was designed to hydroxylate targeted loci and perform site-specific DNA demethylation [15–18].

SLC5A7 is a Na^+^-dependent choline transporter with 13 transmembrane domains, is mainly expressed in the spinal cord and medulla (intracellular vesicles), and belongs to the Na+/glucose co-transporter family (SLC5) [19–21]. SLC5A7 is pivotal in the regulation of cognitive function and neuromuscular junction signaling [22, 23]. Mutations in SLC5A7 have been shown to underlie the dysfunction of cholinergic neurotransmission, which might lead to pathogenesis, including Alzheimer’s disease (AD), myasthenia, cardiovascular disorders, depression, and attention-deficit/hyperactivity disorder (ADHD) [24–27]. In cancer, especially in CRC, the roles and molecular mechanisms of the SLC5 superfamily, including SLC5A7, in tumorigenesis and progression are almost entirely unknown. Ganapathy et al. [28] revealed that SLC5A8 is silenced in several cancer types and is presumed to be a tumor suppressor gene. A study by Koepsell et al. demonstrated that inhibitors of SLC5A1 and SLC5A2 that reduce glucose uptake have shown efficacy in the management of diabetes and cancer [29]. More importantly, our previous findings showed that SLC5A7 expression was decreased in CRC and positively correlated with prognosis [30]. However, the mechanisms underlying SLC5A7 downregulation and its role in CRC remain unclear.

In this study, we aimed to clarify the mechanism underlying the inactivation of SLC5A7 in CRC and the potential correlation between the methylation of the SLC5A7 promoter and prognosis of individuals with CRC. Importantly, we constructed a system based on dCas9-multiGCN4/scFv-TET1CD-sgRNA that targets DNA demethylation at specific loci to reverse SLC5A7 expression level and verified its efficacy and specificity. Moreover, in vitro and in vivo experiments were employed to determine the potential antitumor effect and mechanism of dCas9-multiGCN4/scFv-TET1CD-sgSLC5A7 in CRC. Our results would provide solid evidence for understanding the mechanisms underlying SLC5A7 downregulation and its role in CRC, and a potential therapeutic strategy for CRC, even for other cancers.

## Methods and materials

### Cell lines incubation and antibodies

Human CRC cells (HCT116 and RKO) were obtained from American Type Culture Collection (ATCC, Manassas, VA, USA) and then cultured in high glucose (4.5 g/L) Dulbecco’s modified Eagle’s medium (DMEM, ThermoFisher, Massachusetts, USA) supplemented with 1% penicillin–streptomycin (ThermoFisher, Massachusetts, USA) antibiotics and 10% fetal bovine serum (FBS, ZETA Life, California, USA) at 37 °C in a moisturized atmosphere containing 95% air and 5% CO_2_. All cancer cells were authenticated periodically by DNA profiling on a common platform of the Scientific Research Center, West China Hospital, to ensure that they were mycoplasma-free. Azacitidine (5-azacytidine, AZA, Cat. No. S1782) and decitabine (5-aza-2′-deoxycytidine, DAC, Cat. No. S1200) were purchased from Selleck (Shanghai, China) and used for HCT116 and RKO treatment with a final concentration of 10 μM and 1 μM separately. At 24 h post AZA and DAC treatment, the cells were collected for further analysis.

The antibodies used in this study were as follows: SLC5A7 (MA1-46409, Invitrogen), p53 (10442-1-AP, Proteintech), phospho-p53 (Ser 37; 9289S, CST), p21 (2947, CST), IGFBP3 (10189-2-AP, Proteintech), CDK6 (13331, CST), Bax (2774, CST), and GAPDH (AC002, ABclonal).

### Animal study

Five to six-week-old female BALB/c nude mice were obtained from Gempharmatech Co., Ltd. (Chengdu, China) and allowed to adapt in a SPF condition for a week before use. All animal experiments and procedures were approved by the Institutional Animal Care and Use Committee (IACUC) of West China Hospital, Sichuan University (No. 2021417A), and conformed to IACUC’s regulatory codes. Mice were raised in ventilated cages with a maximal number of 5 mice per cage and provided with adequate water and food. Housing environment was kept at a temperature between 20–24 °C and 30–70% humidified atmosphere. Twelve-hour light–dark cycles were applied to mimic natural day and night shift. To create a subcutaneous cancer model, control sgRNA and sgSLC5A7 HCT116 and RKO cells were subcutaneously implanted into the right flank of nude BALB/c mice (approximately 5 × 10^6^ cells per mouse). Tumor volume (mm^3^) was calculated using the following formula: volume (mm^3^) = width (mm) × length^2^ (mm) × 0.52. Tumors from each mouse were collected after they were killed and weighed.

### Clinical samples and HXCRC cohort

The HXCRC cohort used in the present study contained 69 pairs of malignant tissues and adjacent normal tissues, which were collected from patients with confirmed CRC attending the West China Hospital, Sichuan University, and stored at − 80 °C condition. The overall survival (OS) and disease-free survival (DFS) of patients were recorded for more than 5 years. This part of the study was approved by the Ethics Committee of West China Hospital, Sichuan University (approval number: 2018[280]).

### dCas9-based demethylation vector construction

A vector system (Addgene plasmid 82559), which contains three fundamental components: Cas9 peptide sequence (linker length: 22aa), antibody-sfGFP-TET1CD, and gRNA [16], was used in this study. The gRNA insert fragment was cloned by linearizing the Addgene plasmid 82559 AflII site and Gibson assembly-mediated insertion. The total reaction system combined the AflII-digested plasmid 82559 (50 ng), 1 μL insert oligonucleotide mixture, 6 μL 2 × Gibson Assembly Master Mix, and H_2_O to meet 10 μL in volume. The reaction mixture was then incubated for 15 min at 50 °C. To select the stably transformed clones, competent *Escherichia coli* was combined with 5 μL reaction mixture indicated above and disseminated over a solid medium containing kanamycin. Finally, the plasmid was purified, and the colony was cultured. The targeted sequences were as follows:

- sgSLC5A7-1: CACCGACACCCCTCGCGGTTCCAGG;
- sgSLC5A7-2: CACCGGATACCATGGCTGGCAGCG;
- sgSLC5A7-3: CACCGTGCGGGATGCGAAGGAAACG;
- sgSLC5A7-4: CACCGCCTCGCACCCACACCCCTCG;
- sgSLC5A7-5: CACCGAAAAGTCCCCTTTATAAGGG;
- sgSLC5A7-6: CACCGATGCTCTCCCGGCCCCCTG.

### Cell transfection with sgRNA and small-interfering RNA (siRNA)

For transfection, HCT116 and RKO cancer cells were cultured in 6-well dishes (Corning, New York, USA) and X-tremeGENE HP DNA Transfection Reagent (Roche, Basel, Switzerland) was used following the manufacturer’s protocols for plasmid transfection into cells. G418 (1000 μg/mL) was added to the culture to select successfully transfected cells with a demethylation system. Four days after transfection, the cells were collected to extract genomic DNA and RNA to detect target gene expression and DNA methylation levels. For selecting the stable transfected cells used for animal study, G418 was added to the transfected cells and the concentration was increased from 1000 to 3000 μg/mL, last for 12 days.

The siRNAs for DNMT1, DNMT3a, DNMT3b, and siNC were purchased from Riobio, Co., Ltd., Guangzhou, China. All procedures were consistent with the manufacturer’s protocol, and 48–72 h later, cells were obtained to test the transfection efficiency.

### Quantitative real-time PCR

Whole RNA was extracted from cancer cells using TRIzol reagent (Invitrogen, Carlsbad, CA, USA). Reverse transcription of the extracted RNA into cDNA was achieved using the PrimeScript™ II 1st Strand Synthesis Kit (Takara, Kusatsu, Japan). The acquired complementary DNA replicates were further analyzed using SYBR Green Master Mix (Takara, Kusatsu, Japan) following the reaction conditions of 95 °C (300 s), followed by 45 cycles of 95 °C for 5 s, 58 °C for 30 s, 95 °C for 10 s, 65 °C for 60 s, and 97 °C for 1 s. Quantitative real-time PCR was performed using the QuantStudio3 PCR system (ThermoFisher, Massachusetts, USA), and relative mRNA abundance was calculated using the 2^−ddCt^ method.

### Western blot analysis

Cancer cells were collected after transfection with sgRNA or siRNA. Then, using RIPA lysis buffer (Beyotime, Nanjing, China) containing 1% protease inhibitor cocktail (Merck Millipore, Massachusetts, USA), total protein was extracted on ice for 30 min. Next, the BCA Protein Assay Kit (Thermo Scientific, Massachusetts, USA) was used to determine the protein concentration. Further, using SDS-PAGE gel electrophoresis, 10 μg of extracted proteins with loading buffer was separated and transferred to a PVDF membrane. The PVDF membranes were blocked with 5% skim milk, followed by incubation at 4 °C overnight with antibodies against SLC5A7 (MA1-46409, Invitrogen), p53 (10442-1-AP, Proteintech), phospho-p53 (Ser 37; 9289S, CST), p21 (2947, CST), IGFBP3 (10189-2-AP, Proteintech), CDK6 (13331, CST), Bax (2774, CST), and GAPDH (AC002, ABclonal). The next day, secondary antibodies labeled with horseradish peroxidase (Zsbio, Beijing, China) were incubated for 1 h, and visualization was performed using an ECL western blotting substrate kit (ThermoFisher, Massachusetts, USA).

### Analysis of DNA methylation

A Tissues & Cell Genomic DNA Purification Kit (DP021, GeneMark) was used to extract genomic DNA from cells and samples, and the modified DNA was used for time-of-flight mass spectrometry, which was performed by Oebiotech Co., Ltd., Shanghai, China.

### Cell proliferation and colony formation assays

Cell Counting Kit 8 (CCK-8; Dojindo, Kumamoto, Japan) was used to measure cell viability. In brief, approximately 1000 cancer cells per well, with five replicates, were seeded in 96-well plates and incubated. After 72 h of incubation, 10 μL of CCK-8 reagent was added to the culture and incubated for 3–4 h. Then, the absorbance at 450 nm was measured for each well. In the colony-formation assay experiments, approximately 1000 cells per well were seeded in triplicate in six-well plates and incubated for 8–10 days. When visible cell colonies were detected, each well was gently washed twice with phosphate-buffered saline (PBS) and then fixed with 4% paraformaldehyde. Finally, after staining with 0.1% crystal violet, the visible colonies were counted under an inverted microscope. Each experiment was repeated at least three times.

### Immunohistochemistry (IHC) staining

IHC staining of paraffin-embedded tissues of subcutaneous tumors was performed with primary antibodies against SLC5A7 (MA1-46409, Invitrogen), p53 (10442-1-AP, Proteintech), and phospho-p53 (Ser 37; 9289S, CST) in a moist dark chamber at 4 °C for 8–10 h. Then, these specimens were incubated with secondary antibody for approximately 2 h and stained with DAB (Maixin, Fuzhou, China). Positive cells were deemed to be cells with moderate to strong brown staining in both the nucleus and cytoplasm.

### Statistical analysis

All experiments were conducted 3–5 times to ensure a consistent outcome. Kaplan–Meier survival analysis of disease-free survival (DFS) and overall survival (OS) was performed using the median value as the cutoff point, and significance comparison was examined using the log-rank test. All data are presented as the mean ± SD. Two-tailed unpaired Student’s *t* and Chi-square tests were used for further comparisons, and Pearson’s correlation analysis was performed to determine the correlation between SLC5A7 expression and methylation level. Statistical significance was set at *p* ≤ 0.05. All statistical analyses were performed using the GraphPad Prism 5 (California, USA).

## Result

### DNA methylation mediates SLC5A7 downregulation in CRC

**Fig. 1:**
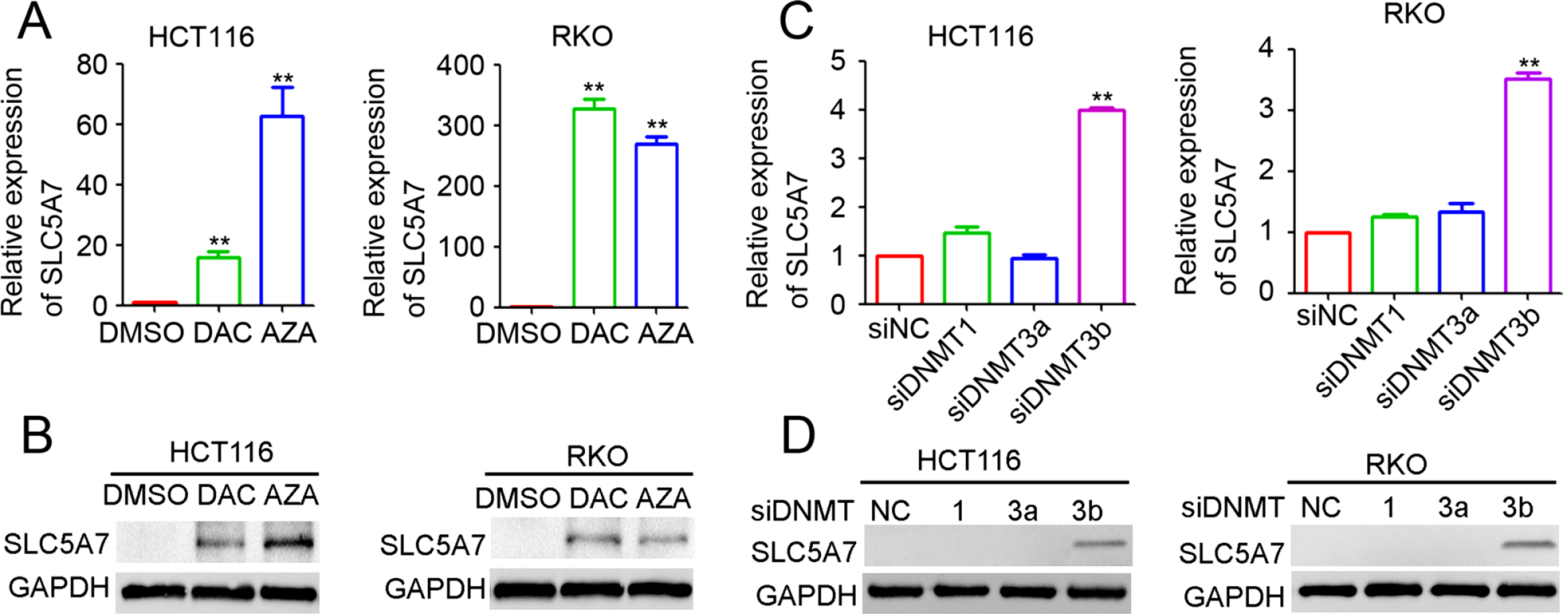
SLC5A7 downregulation is modulated by DNA methylation in CRC. **A** SLC5A7 mRNA expression levels in HCT116 and RKO after DAC and AZA treatment (**p < 0.01, compared with the DMSO group). **B** SLC5A7 protein expression levels in HCT116 and RKO after DAC and AZA treatment, GAPDH was used as a loading control. **C** SLC5A7 mRNA expression levels in HCT116 and RKO after transfection of siNC, siDNMT1, siDNMT3a, siDNMT3b (*p* < 0.01, compared with the siNC group). **D** SLC5A7 protein expression levels in HCT116 and RKO after transfection of siNC, siDNMT1, siDNMT3a, siDNMT3b, GAPDH was used as a loading control

Our previous study showed that SLC5A7 is downregulated in the majority of CRC tissues and cells [30]. To further reveal the potential mechanism underlying the inactivation of SLC5A7 in CRC, two broad-spectrum demethylation chemicals DAC and AZA were utilized to treat HCT116 and RKO cells. Higher SLC5A7 mRNA and protein expression levels were observed after DAC and AZA intervention compared to the untreated control group in both HCT116 and RKO cells (Fig. 1 A&B). To further investigate the role of methylation in SLC5A7 inactivation in CRC cells, siRNA targeting DNMT1, DNMT3a, and DNMT3b was used and shown to efficiently inhibit DNMT1, DNMT3a, and DNMT3b expression (Additional file 1: Fig. S1). Our results indicated that DNMT3b was involved in the specific methylation of SLC5A7, because SLC5A7 mRNA and protein expression was upregulated followed by silencing of DNMT3b (Fig. 1C, D), whereas silencing of DNMT1 and DNMT3a expression had no significant effect on SLC5A7 expression in CRC cells (Fig. 1C, D). Collectively, these results suggest that the expression of SLC5A7 is suppressed by DNMT3b-mediated DNA methylation in CRC cells.

### SLC5A7 DNA promoter methylation is inversely correlated with SLC5A7 expression and is a prognostic biomarker for CRC patients

**Fig. 2:**
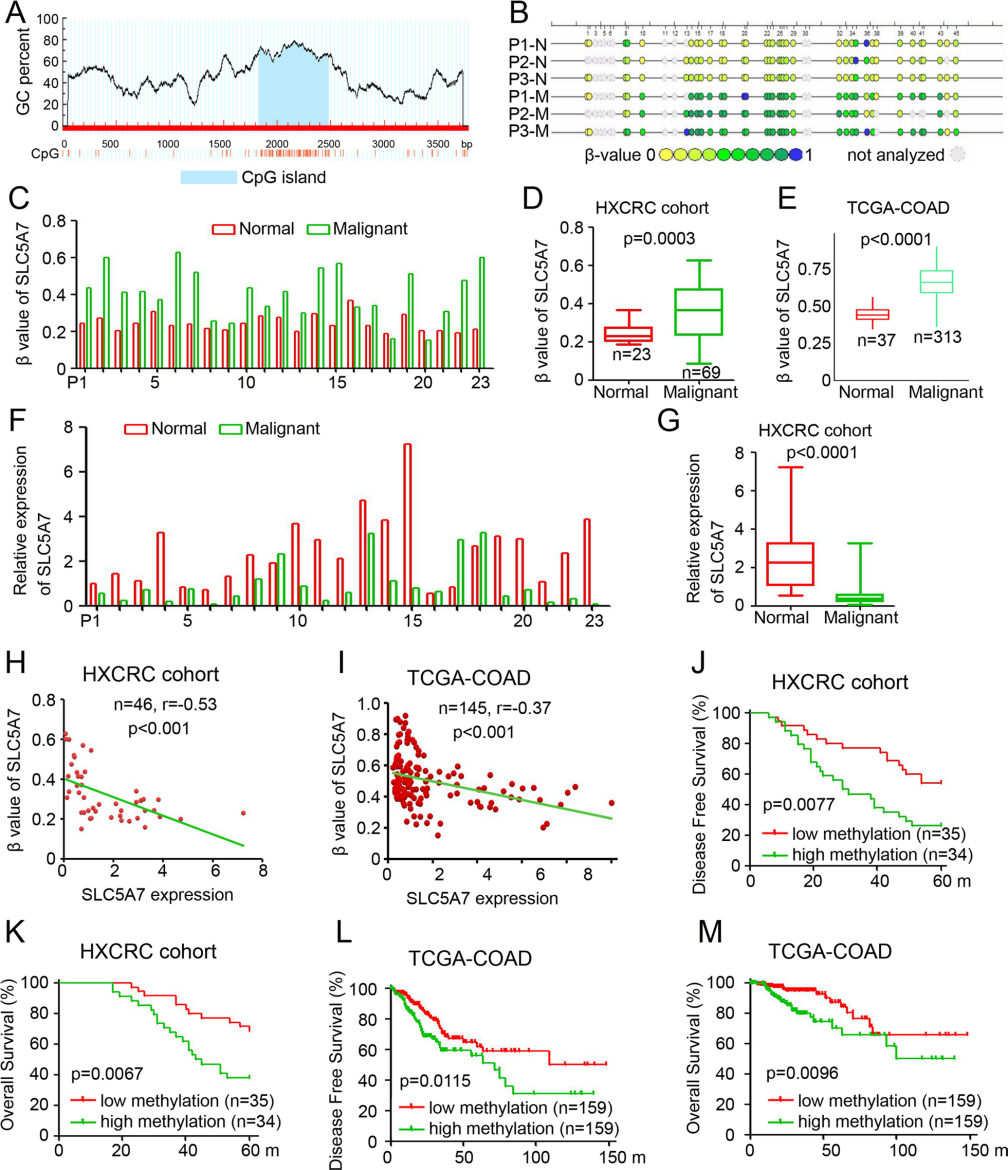
SLC5A7 methylation status and its correlation with SLC5A7 expression and prognosis of CRC patients. **A** DNA sequencing analysis showed that CpG island was enriched in 1800–2500 bp that can be potentially methylated. **B** Determination of SLC5A7 methylation in CRC tissues and paired normal colorectal tissues by time-of-flight mass spectrometry. The dots represent CpG sites and the color of the bar below from green to blue indicates *β* value range from 0 to 1. **C** SLC5A7 promotor methylation in 23 paired CRC tissues. **D**, **E** SLC5A7 methylation level analysis in tumor adjacent tissues and malignant tissues in HXCRC cohort (**D**) and TCGA-COAD cohort (**E**). **F** SLC5A7 mRNA expression in 23 paired CRC tissues. **G** SLC5A7 expression analysis in tumor adjacent tissues and malignant tissues in HXCRC cohort. **H**, **I** Correlation analysis between SLC5A7 methylation level and mRNA expression in HXCRC cohort (Pearson correlation, *r* = − 0.53, *p* < 0.001, **H**) and TCGA-COAD cohort (r = − 0.53, *p* < 0.001, **I**). **J**, **K** Kaplan–Meier analysis showing the disease-free survival of colon cancer patients in HXCRC cohort stratified by median SLC5A7 methylation level (*n* = 93; *p* = 0.0077; long-rank test, **J**), and overall survival (*n* = 93; *p* = 0.0067; long-rank test, **K**). **L**, **M** Kaplan–Meier analysis showing the disease-free survival of colon cancer patients in TCGA-COAD cohort stratified by median SLC5A7 methylation level (*n* = 318; *p* = 0.0115; long-rank test, **L**), and overall survival (*n* = 318; *p* = 0.0096; long-rank test, **M**)

To further investigate the role of DNA methylation in SLC5A7 inactivation in CRC cells, DNA sequence analysis of SLC5A7 was performed. Our results showed that CpG island 1800–2500 bp upstream of SLC5A7 was enriched, which could be potentially methylated (Fig. 2A). Time-of-flight mass spectrometry was used to determine the methylation status of the SLC5A7 sequence (1800–2500 bp) in 69 CRC tissues from the HXCRC cohort. Our results demonstrated a higher methylation percentage of the SLC5A7 DNA promoter in malignant samples than in normal tissues in the HXCRC cohort (Fig. 2B–D). Similar results were confirmed using the TCGA-COAD database (Fig. 2E). Furthermore, compared to normal adjacent tissues, qPCR results demonstrated that SLC5A7 was downregulated in most human CRC tissues (Fig. 2F, G). Indeed, the SLC5A7 methylation β value was negatively correlated with its expression in the HXCRC and TCGA-COAD cohorts, with r values of − 0.53 (*p* < 0.001, Fig. 2H) and − 0.37 (*p* < 0.001, Fig. 2I), respectively. Moreover, the SLC5A7 methylation level in both cohorts was determined as “high” or “low” according to the median value. The results revealed that SCL5A7 was a negative predictor of DFS and OS in the HXCRC cohort (*n* = 69, *p* = 0.0077 and *p* = 0.0067, respectively) and TCGA-COAD cohort (*n* = 318, *p* = 0.0115 and *p* = 0.0096, respectively) (Fig. 2J-M). In summary, DNA methylation of SLC5A7 is inversely correlated with its expression and CRC patient prognosis.

### Targeted and specific demethylation of SLC5A7 in CRC cells with dCas9-multiGCN4/scFv-TET1CD-sgRNA transfection

**Fig. 3:**
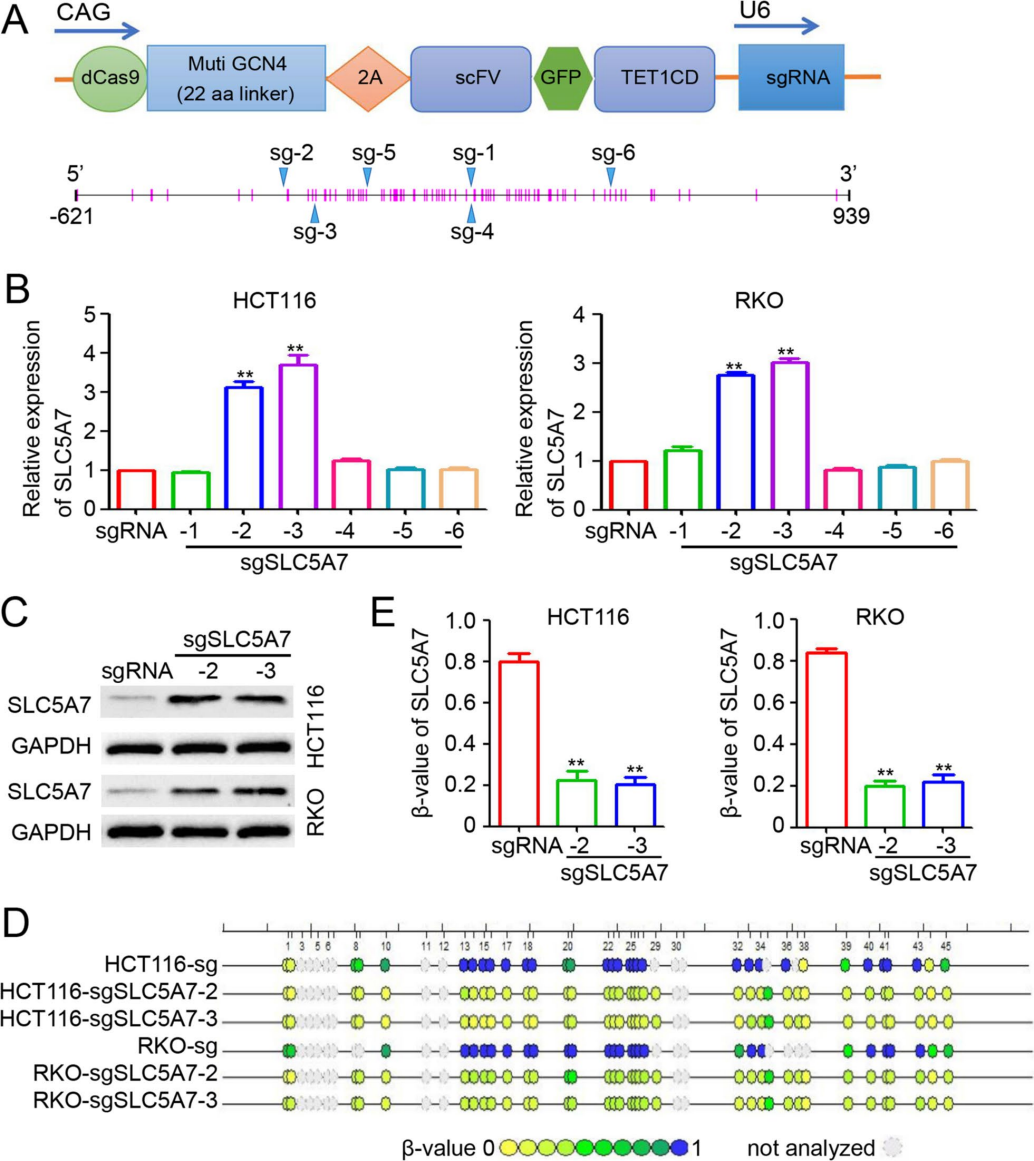
Demethylation of SLC5A7 by dCas9 and sgSLC5A7. **A** Arrangement of specific demethylation system dCas9-multiGCN4/scFv-TET1CD-sgRNA. The target sites of sgRNAs are illustrated in blue arrowheads. **B** SLC5A7 mRNA expression determined by qPCR after transfection of dCas9-multiGCN4/scFv-TET1CD-sgRNA and sgSLC5A7(1–6). (Transfection of dCas9-multiGCN4/scFv-TET1CD-sgRNA have only used dCas9-multiGCN4/scFv-TET1CD fragment which do not specific target SLC5A7 site.) **C** Western blot results showed that SLC5A7 protein expression elevated after transfection of sgSLC5A7-2 and sgSLC5A7-3. **D**, **E** DNA methylation analysis of selected CpG sites in SLC5A7 gene after using sgSLC5A7-2 and sgSLC5A7-3 targeted demethylation reagents by time-of-flight mass spectrometry. The dots represent CpG islands and the color of the bar below from green to blue indicates *β* value range from 0 to 1 (**D**). SLC5A7 promotor methylation was analyzed (**p < 0.01, compared with sgRNA group, **E**)

For targeting the SLC5A7 promoter region from − 621 to 939 bp, six sgRNAs were designed to construct dCas9-multiGCN4/scFv-TET1CD-sgRNAs, which could specifically demethylate the SLC5A7 gene to reverse its expression [16] (Fig. 3A). After the transfection of dCas9-multiGCN4/scFv-TET1CD-sgSLC5A7, significant increases in SLC5A7 transcription were observed in HCT116 and RKO cells transfected with sgSLC5A7-2 and -3 (Fig. 3B). Thus, sgSLC5A7-2 and sgSLC5A7-3, which exert the most significant effects, were used for further analyses. Western blot results confirmed the effective upregulation of SLC5A7 in HCT116 and RKO cells (Fig. 3C). Furthermore, time-of-flight mass spectrometry revealed reductions in CpG methylation in sgSLC5A7-2- and sgSLC5A7-3-transfected CRC cells (Fig. 3D, E). Concerns about the specificity in the use of targeted modulation methods are increasing, and off-target effects of Cas9-based techniques seem to be a major hindrance in its practical usage. Therefore, the top potential off-target loci were analyzed using a web-based tool (https://crispr.bme.gatech.deu/) [31] to determine whether their mRNA transcription was changed due to off-target demethylation by sgSLC5A7-2 (Additional file 2: Fig. S2A) and sgSLC5A7-3 (Additional file 2: Fig. S2B). As shown in Additional file 2: Fig. S2C and D, no meaningful mRNA expression disparities were observed among these potential off-target genes between the SLC5A7-2 and sgRNA groups (Additional file 2: Fig. S2C) or between SLC5A7-3 and sgRNA groups (Additional file 2: Fig. S2D). In general, these data verified that dCas9-based sgSLC5A7-2 and sgSLC5A7-3 were efficient and specific in SLC5A7 demethylation.

### Targeted demethylation of SLC5A7 impairs CRC growth both in vitro and in vivo

**Fig. 4:**
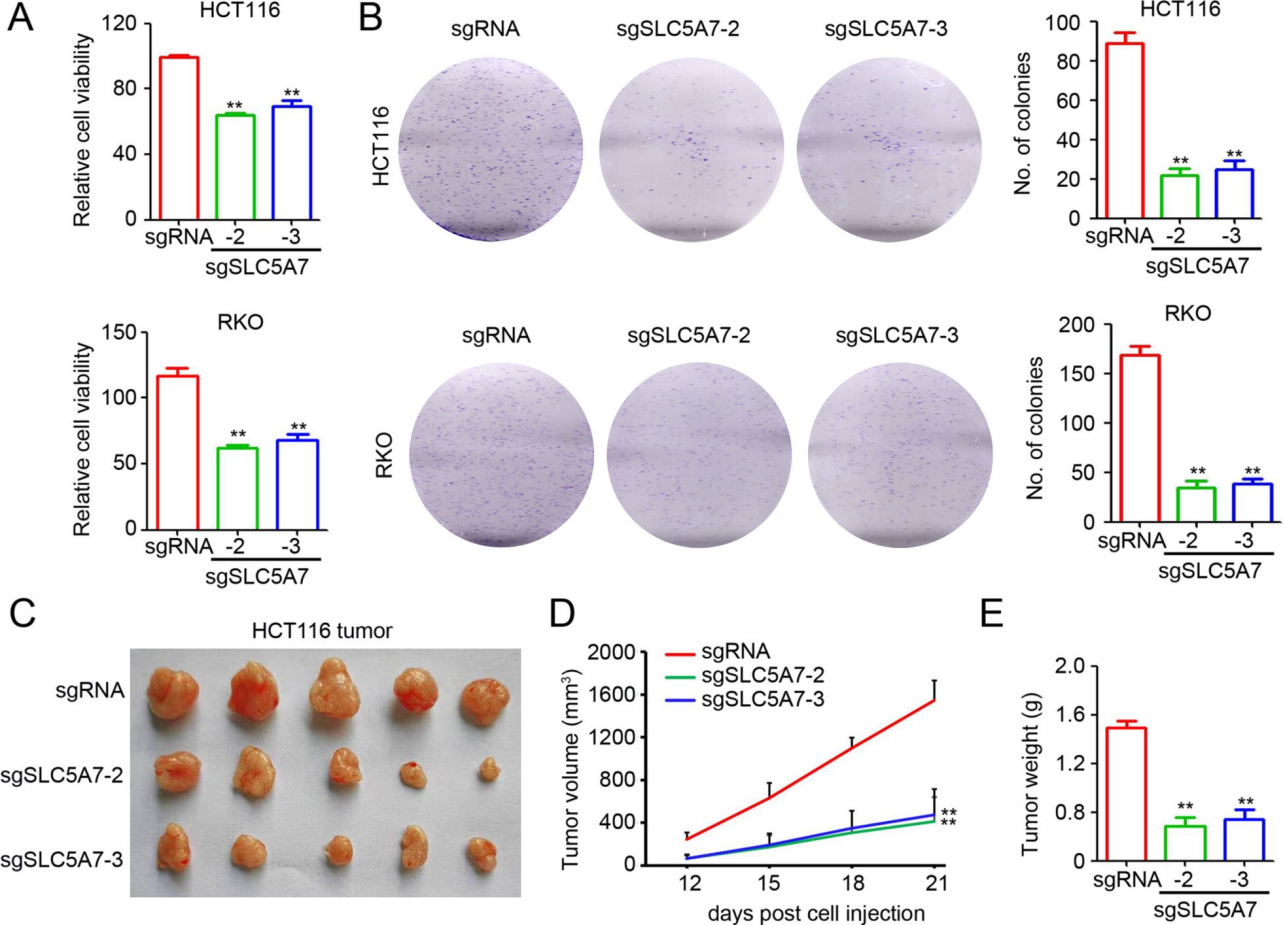
Anti-tumor effect of targeted demethylation of SLC5A7 in vitro and in vivo. **A** Cell counting kit-8 (CCK-8) assay was applied to detect cell viability after transfection of sgRNA, sgSLC5A7-2, and sgSLC5A7-3 (*n* = 5; **p < 0.01, Student’s *t* test). **B** Clone formation assay was performed to detect the ability of tumor colony formation after transfection of sgRNA, sgSLC5A7-2, and sgSLC5A7-3. Each well incubated with approximately 1000 cancer cells (*n* = 3; **p < 0.01, Student’s *t* test). **C** Macroscopic images of stable transfection of sgRNA, sgSLC5A7-2, and sgSLC5A7-3 HCT116 tumors. **D**, **E** Tumor volume (**D**) at different time point and tumor weight (**E**) at killing were monitored. Approximately 5 × 10^6^ HCT116 cells stably transfected with sgRNA, sgSLC5A7-2, and sgSLC5A7-3 were injected in the right flank of female BALB/c nude mice (**p < 0.01, Student’s *t* test)

Next, to examine the possible anti-tumor effect of the selective de-methylation of SLC5A7 in CRC, further in vitro and in vivo experiments were performed using dCas9-based sgSLC5A7-2- and sgSLC5A7-3-transfected CRC cells. These results revealed that lower tumor cell viability and colony formation resulted from the transfection of sgSLC5A7-2 and sgSLC5A7-3 into HCT116 and RKO cancer cells (Fig. 4A, B). Furthermore, a subcutaneous tumor model with an injection of approximately 5 × 10^6^ HCT116 cells into the right flank of female BALB/c nude mice showed that HCT116 cells stably transfected with sgSLC5A7-2 and sgSLC5A7-3 had significantly smaller tumor volume (Fig. 4C, D) and weight (Fig. 4E) compared to those transfected with sgRNA. In summary, these results confirmed the anti-tumor effects of the targeted demethylation system with sgSLC5A7-2 and sgSLC5A7-3 in CRC cells.

### Targeted demethylation of SLC5A7 regulates p53 and its downstream target expression

**Fig. 5:**
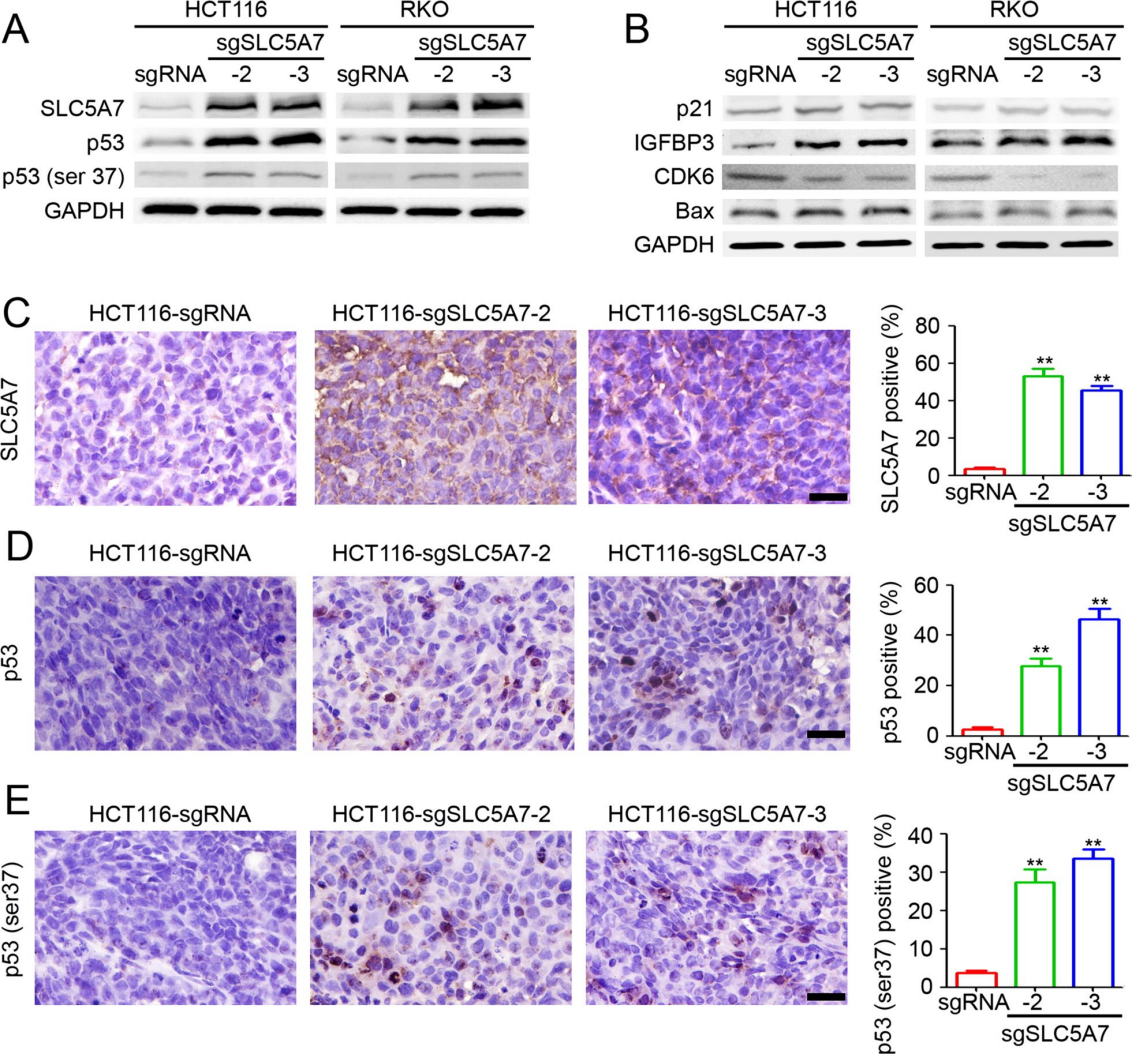
Targeted demethylation of SLC5A7 influence of p53 and its downstream targets. **A** Western blot results for protein expression of SLC5A7, p53, p53 (ser37) and GAPDH in HCT116 and RKO tumors stably transfected with sgRNA, sgSLC5A7-2, and sgSLC5A7-3. **B** p21, IGFBP3, CDK6, Bax and GAPDH expression level in HCT116 and RKO tumors stably transfected with sgRNA, sgSLC5A7-2, and sgSLC5A7-3. **C**–**E** Immunohistochemical (IHC) staining of SLC5A7 (**C**), p53 (**D**), and p53 (ser37) (**E**) in HCT116 tumors. The percentages of SLC5A7, p53, and p53 (ser37) positive cells were calculated and compared with sgRNA control group (*n* = 5, **p < 0.01, Student’s *t* test)

In our previous study, we demonstrated that the expression of SLC5A7 was involved in the p53 signaling pathway and stabilization of p53 [30]. The present results confirmed that the implementation of targeted demethylation was also associated with higher p53 and phosphor-p53 (ser 37) expression in cancer cells (Fig. 5A). Furthermore, western blot results confirmed the upregulation of p21, IGFBP3, and Bax levels and inverse regulation of CDK6 expression in sgSLC5A7-2 and sgSLC5A7-3 CRC cells (Fig. 5B). Additionally, IHC staining of subcutaneous xenograft HCT116 tumors transfected with sgSLC5A7-2 and sgSLC5A7-3 indicated increased SLC5A7 (Fig. 5C), p53 (Fig. 5D), and phospho-p53 (ser 37) (Fig. 5E) expression levels compared to the sgRNA group. Taken together, these results suggest that targeted demethylation of SLC5A7 can be utilized to modulate and stabilize p53 levels by directly upregulating the expression of SLC5A7.

## Discussion

Our results revealed that SLC5A7 promotor methylation mediated the inactivation of SLC5A7 in CRC, and that SLC5A7 methylation can be utilized as a valuable biomarker for predicting the prognosis of CRC patients. In addition, we constructed an efficient and specific demethylation system, dCas9-multiGCN4/scFv-TET1CD-sgSLC5A7, which could significantly increase SLC5A7 expression in CRC cells by demethylating the SLC5A7 promoter. Furthermore, the anti-tumor effect of dCas9-multiGCN4/scFv-TET1CD-sgSLC5A7 via modulation and stabilization of p53 was confirmed in vitro and in vivo.

The majority of DNA methylation occurs on cytosines or CpG sites, and it is believed that methylated CpG-rich regions (known as CpG islands) at transcription start sites inhibit subsequent gene transcription [32, 33]. Indeed, methylation of suppressor gene regions is thought to participate in carcinogenesis [34]. Application of targeted DNA demethylation successfully increased the expression of genes that were repressed. Therefore, DNA demethylation can be developed as a potential target for cancer therapy [4]. In our previous study, SLC5A7 was downregulated in CRC, and its expression was correlated with prognosis, which is consistent with a previous study indicating a similar trend of SCL5A7 in several other cancer types [35]. In the present study, we demonstrated that SLC5A7 was hypermethylated and by using two broad-spectrum demethylation agents (DAC and AZA), the expression of SLC5A7 was restored in CRC cells. Using the dCas9-targeted demethylation system, we found that sgSLC5A7-2 and sgSLC5A7-3 exhibited the strongest effect of demethylating sgSLC5A7, followed by a significant upregulation of SLC5A7 expression. Furthermore, inverse correlations between SLC5A7 expression and SLC5A7 methylation levels were observed in malignant colon cancer tissues. In addition, prognosis assessment demonstrated that lower SLC5A7 methylation indicated a longer DFS and OS in CRC patients. Accordingly, DNA methylation plays an important role in regulating SLC5A7 expression in cancer tissues.

Because of some undesirable side effects resulting from the application of global demethylation agents such as AZA and DAC, more precise and effective strategies for demethylation are needed. Thus, the dCas9-multiGCN4/scFv-TET1CD-sgRNA-based system that targets the selected DNA methylated loci was applied. There are also other methods such as ZFs [9] and TALE [10]-based tools; however, they are less efficient and convenient [16]. Our results showed that dCas9-mul-tiGCN4/scFv-TET1CD-sgSLC5A7-2 and -3 could effectively reverse the downregulation of SLC5A7 by targeting its methylated promoter region. Moreover, we detected possible off-target effects of dCas9-mul-tiGCN4/scFv-TET1CD- sgSLC5A7-2 and -3 on other sites using a web-based system [31] and demonstrated that dCas9-multiGCN4/scFv-TET1CD-sgSLC5A7 was effective and specific for targeted DNA demethylation.

Choline is crucial in the formation and biological function of cell membranes and cholinergic neurotransmission, because it is a fundamental resource of phospholipid phosphatidylcholine and the synthesis of the neurotransmitter acetylcholine (ACh) [22, 36]. SLC5A7 participates in the absorption of choline from the synaptic cleft after neurotransmitter release and exerts time-limiting effects in ACh synthesis, which are reported to regulate cell growth [22]. However, studies have focused on the role of SLC5A7 in cancer development, and its expression level varies among different types of tumors. Penet and Eliyahu et al. [37, 38] found overexpression of SLC5A7 in pancreatic ductal adenocarcinoma and breast cancer due to their high choline metabolism. In contrast, Li et al. [35] showed that SLC5A7 expression was downregulated in several cancer types, including thyroid cancer (THCA) and pheochromocytoma and paraganglioma (PCPG). Yang and colleagues [39] revealed that SLC5A7 was hypermethylated and downregulated in lung adenocarcinoma (LUAD), but not in lung squamous cell carcinoma (LUSC). These disparities might be due to the distinct metabolic profiles of the different types of tumors. In the present study, we found that with the upregulation of SLC5A7 by the targeted demethylation system, the expression of tumor suppressor gene p53 increased and phosphor-p53 (ser 37), which inhibits MDM2 binding and p53 degeneration, was also upregulated [40, 41]. In vivo and in vitro experiments also demonstrated that targeted demethylation of the SLC5A7 promoter can strongly inhibit tumor proliferation and progression, and these effects might be mediated by increased SLC5A7 expression. Furthermore, other downstream targets of p53, such as p21, IGFBP3, and Bax, were upregulated, and CDK6 was inhibited after transfection with sgSLC5A7-2 and sgSLC5A7-3. Based on these results, we hypothesized that targeted demethylation of SLC5A7 could stabilize p53 and cause p53-mediated inhibition of the cancer cell cycle and enhancement of apoptosis [40], which would be a targeted therapeutic strategy for CRC. However, mutant p53 actively promotes the development of cancer through gain-of-function activities [42, 43]. And our previous study indicated that SLC5A7 had little effect on cancer cell proliferation in CRC cells expressing mutant p53 [30]. Thus, the potential therapeutic application of targeted demethylation of SLC5A7 may depend on p53 mutation status.

## Conclusions

In conclusion, our results demonstrated that the DNA methylation level mediates SLC5A7 expression and could be used as a prognostic indicator for CRC patients. Next, by using dCas9-multiGCN4/scFv-TET1CD-sgSLC5A7 that specifically demethylates the SLC5A7 promoter region, the expression of SLC5A7 could be restored. The implementation of targeted SLC5A7 DNA demethylation reagents resulted in significant anti-tumor effects both in vitro and in vivo. These anti-tumor effects have been demonstrated to be associated with the stabilization of p53 and its downstream regulators. Moreover, the dCas9-multiGCN4/scFv-TET1CD-sgSLC5A7 may have promising therapeutic value in colon cancer and other cancers.

## Supplementary Information

**Additional file 1: Fig. S1.** Specificity of siDNMT1, siDNMT3a, and siDNMT3b. (A) Expression determination of DNMT1 after transfection of siNC, siDNMT1, siDNMT3a, and siDNMT3b in HCT116 cells by qPCR (*n* = 3; **p < 0.01, compared with siNC group). (B) Expression determination of DNMT3a after transfection of siNC, siDNMT1, siDNMT3a, and siDNMT3b in HCT116 cells by qPCR (*n* = 3; **p < 0.01, compared with siNC group). (C) Expression determination of DNMT3b after transfection of siNC, siDNMT1, siDNMT3a, and siDNMT3b in HCT116 cells by qPCR (*n* = 3; **p < 0.01, compared with siNC group).

**Additional file 2: Fig. S2.**. Off-target analysis of the dCas9-based specific demethylation system. (A&B) 8 and 5 potential off-target sites predicted for sgSLC5A7-2 and sgSLC5A7-3, respectively, using a web-based system as preciously described [31]. (C&D) qPCR was employed to detect the mRNA expression analysis of predicted off-target gene that might be influenced by sgSLC5A7-2 and sgSLC5A7-3, compared with the sgRNA group. Data are shown after normalization to GAPDH.

## Data Availability

Data related to gene expression, DNA methylation, and clinical information of TCGACOAD are available from GDC data portal (https://portal.gdc.cancer.gov).

## Abbreviations

SLC5A7: Solute carrier family 5 member 7
CHT1: Choline transporter 1
CRC: Colorectal cancer
AZA: Azacitidine/5-azacytidine
DAC: Decitabine/5-aza-2′-deoxycytidine
DNMTis: DNA methyltransferase inhibitors
TALE: Transcription activator-like effector
TET1: Ten eleven translocation 1
AD: Alzheimer’s disease
ADHD: Attention-deficit/hyperactivity disorder
IACUC: Institutional Animal Care and Use Committee
IHC: Immunohistochemistry
TCGA: The Cancer Genome Atlas
COAD: Colon adenocarcinoma
