# Supplementary figures and images for: Targeted demethylation of the SLC5A7 promotor inhibits colorectal cancer progression
Source: Clin Epigenetics. 2022 Jul 20;14:92. doi: 10.1186/s13148-022-01308-5 (PMC9301853; doi:10.1186/s13148-022-01308-5)

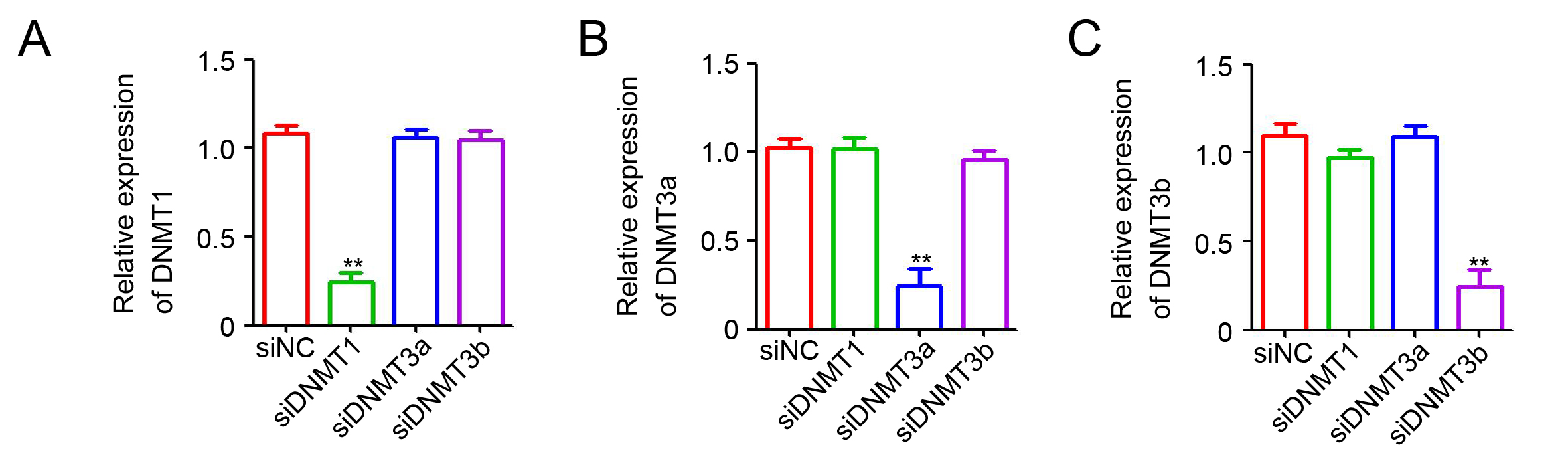

Supplement: Supplementary file 1 — Additional file 1: Fig. S1. Specificity of siDNMT1, siDNMT3a, and siDNMT3b. (A) Expression determination of DNMT1 after transfection of siNC, siDNMT1, siDNMT3a, and siDNMT3b in HCT116 cells by qPCR (n = 3; **p < 0.01, compared with siNC group). (B) Expression determination of DNMT3a after transfection of siNC, siDNMT1, siDNMT3a, and siDNMT3b in HCT116 cells by qPCR (n = 3; **p < 0.01, compared with siNC group). (C) Expression determination of DNMT3b after transfection of siNC, siDNMT1, siDNMT3a, and siDNMT3b in HCT116 cells by qPCR (n = 3; **p < 0.01, compared with siNC group). [file 13148_2022_1308_MOESM1_ESM.jpg]

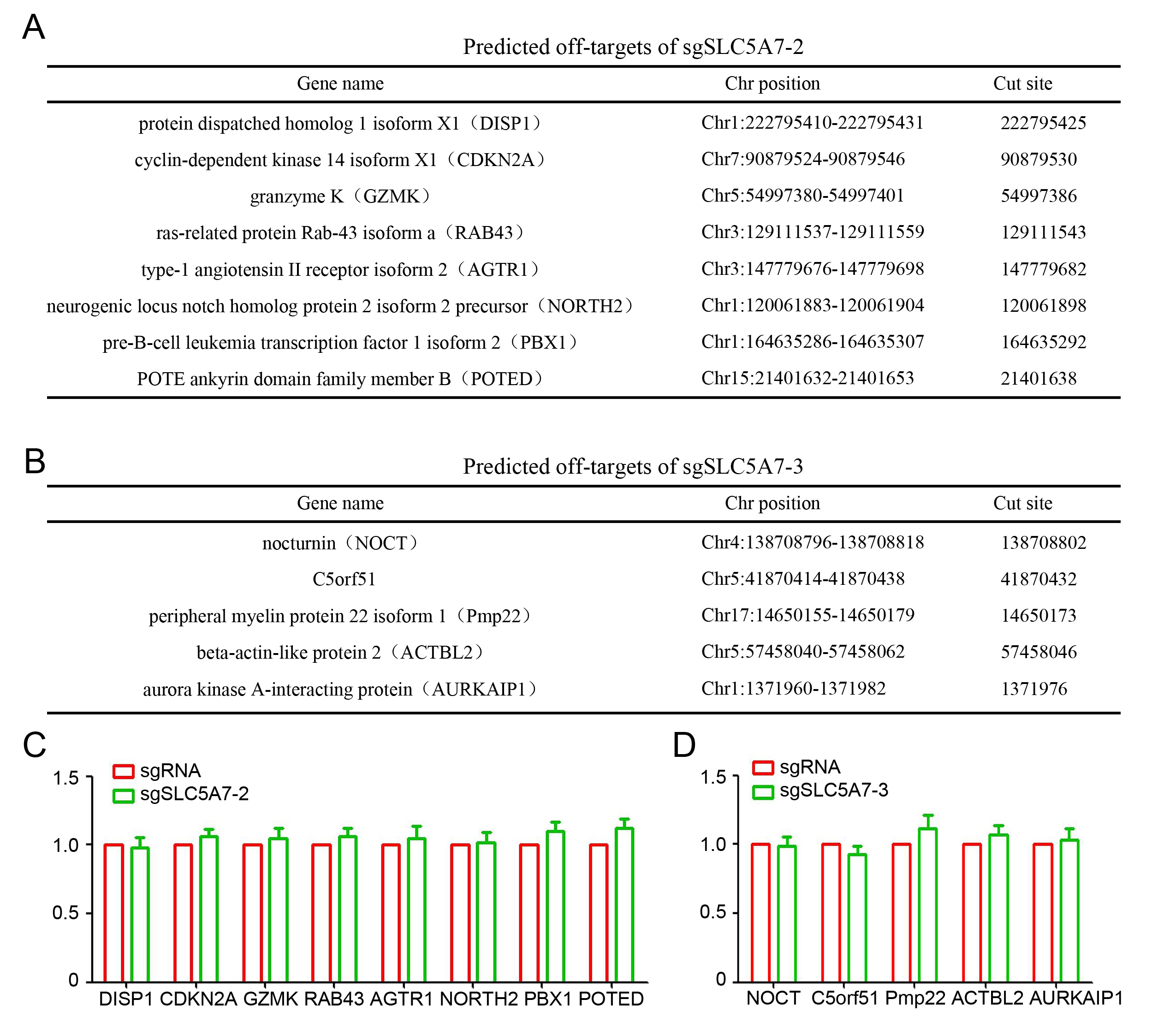

Supplement: Supplementary file 2 — Additional file 2: Fig. S2.. Off-target analysis of the dCas9-based specific demethylation system. (A&B) 8 and 5 potential off-target sites predicted for sgSLC5A7-2 and sgSLC5A7-3, respectively, using a web-based system as preciously described [31]. (C&D) qPCR was employed to detect the mRNA expression analysis of predicted off-target gene that might be influenced by sgSLC5A7-2 and sgSLC5A7-3, compared with the sgRNA group. Data are shown after normalization to GAPDH. [file 13148_2022_1308_MOESM2_ESM.jpg]
